# Integration of single-cell RNA and bulk RNA sequencing revealed malignant ductal cell heterogeneity and prognosis signatures in pancreatic cancer

**DOI:** 10.3389/fimmu.2025.1579184

**Published:** 2025-07-01

**Authors:** Haiyang Du, Gao Si, Jiqing Si, Xuejie Song, Fuchun Si

**Affiliations:** ^1^ Henan University of Chinese Medicine, Traditional Chinese Medicine (Zhongjing) School, Zhengzhou, China; ^2^ Henan University of Chinese Medicine, Henan Key Laboratory of Traditional Chinese Medicine (TCM) Syndrome and Prescription Signaling, Henan International Joint Laboratory of Traditional Chinese Medicine (TCM) Syndrome and Prescription Signaling, Academy of Chinese Medical Sciences, Zhengzhou, China; ^3^ Department of Orthopedic, The Third Hospital of Peking University, Beijing, China; ^4^ Henan Hospital of Traditional Chinese Medicine (TCM), The Second Affiliated Hospital of Henan University of Traditional Chinese Medicine, Zhengzhou, China

**Keywords:** pancreatic cancer, tumor microenvironment, CTSV, macrophages, CXCL14-CXCR4

## Abstract

**Introduction:**

Pancreatic cancer is a highly malignant tumor of the digestive system with a dismal prognosis. Despite advances in diagnosis and treatment, overall survival remains extremely low. Early diagnostic markers and an improved understanding of tumor-microenvironment interactions are essential for developing more effective therapies.

**Methods:**

We analyzed 74 single-cell RNA sequencing (scRNA-seq) samples, performing unsupervised clustering and marker-gene expression profiling to define major cell types. Large-scale chromosomal copy-number variation (CNV) analysis distinguished malignant from non-malignant ductal cells. Non-negative matrix factorization (NMF) identified stage-associated gene modules, which were integrated with TCGA bulk-RNA data and machine-learning feature selection to pinpoint candidate prognostic genes. Two independent cohorts were used for validation. Regulatory network inference (pySCENIC) and ligand–receptor interaction analysis (CellPhoneDB) explored cross-talk between malignant cells and macrophages. Finally, in vitro knockdown of CTSV assessed its functional role in pancreatic cancer (PAC) cell proliferation and migration.

**Results:**

Three prognosis-related genes—ANLN, NT5E, and CTSV—were selected based on their strong association with clinical stage and validated in external datasets. High expression of these genes correlated with poorer overall survival and an increased infiltration of M0 macrophages. CellPhoneDB predicted significant interactions between high-expression malignant ductal cells and M0 macrophages via CXCL14–CXCR4 and IL1RAP–PTPRF axes, with SPI1 identified as an upstream regulator of IL1RAP. In vitro CTSV knockdown significantly inhibited PAC cell proliferation and migration.

**Discussion:**

Our integrative single-cell and bulk-RNA workflow identifies ANLN, NT5E, and CTSV as novel prognostic biomarkers in pancreatic cancer and highlights a pro-tumorigenic interaction between malignant ductal cells and macrophages. Targeting CTSV or disrupting CXCL14–CXCR4 and IL1RAP–PTPRF signaling may offer new therapeutic avenues for PAC.

## 1 Introduction

Pancreatic cancer (PAC) is a highly malignant tumor of the digestive system with a poor prognosis. In recent years, its incidence and mortality rates have been on the rise, making it the third leading cause of cancer-related death (1). Due to the lack of obvious early symptoms, most patients are diagnosed at an advanced stage, making surgical resection opportunities extremely limited (2, 3). Even with surgery, the recurrence rate of PAC remains high, with a five-year survival rate of less than 10% (1). Currently, the main treatment options for PAC include surgery, chemotherapy, and radiotherapy, but their effectiveness remains unsatisfactory. In recent years, the successful application of immunotherapy in various cancers has attracted widespread attention (4), but its efficacy in PAC is limited, which is closely related to the complex tumor immune microenvironment of PAC (5, 6). Therefore, there is an urgent need to study the immune microenvironment of PAC in depth to identify new early diagnostic markers and therapeutic targets.

The rise of single-cell RNA sequencing (scRNA-seq) technology provides a new perspective for studying the cellular heterogeneity within the tumor microenvironment (TME) (7, 8). For instance, early scRNA-seq studies by Elyada et al. (9)and Peng et al. (10) comprehensively profiled tumor and stromal cell populations in PAC, revealing heterogeneous subtypes of cancer-associated fibroblasts and identifying distinct malignant ductal cell states. These findings illuminated how fibroblast diversity and ductal heterogeneity contribute to disease progression and may modulate antitumor immune responses—particularly through interactions with T cells. However, while these works (9, 10) established a baseline understanding of stromal and malignant heterogeneity in PAC, they did not fully address the functional interplay between malignant ductal cells and immune populations such as macrophages. Recent scRNA-seq studies have begun to explore the immunological landscape of PAC in greater depth: for example, Hwang et al. (11) characterized multicellular dynamics in response to neoadjuvant therapy, uncovering how tumor cells and immune components co-evolve under treatment pressure. Nonetheless, the specific molecular crosstalk between malignant ductal subpopulations and myeloid cells remains incompletely understood, particularly with regard to how these interactions might drive tumor progression and shape clinical outcomes.

In this study, we address these gaps by integrating scRNA-seq data from 74 samples (68 pancreatic cancer patients) and publicly available bulk RNA-seq data from The Cancer Genome Atlas (TCGA). First, we aim to characterize novel malignant ductal subpopulations. Second, we seek to uncover key ligand–receptor interactions—particularly those involving macrophages—via single-cell regulatory network inference. Finally, and most importantly, we strive to identify robust prognostic genes through machine-learning approaches. By achieving these goals, we hope to deepen our understanding of the PAC TME, lay the groundwork for early diagnostic markers and targeted therapeutic strategies, and thereby potentially guide risk stratification and therapeutic decisions for PAC patients.

## 2 Materials and methods

### 2.1 Data collection and preprocessing

In this research, we acquired scRNA-seq data for PAC from multiple databases. Specifically, dataset PRJNA878527, which includes 6 tumor and 6 normal samples, was downloaded from the European Nucleotide Archive (ENA) (accessed June 24, 2024), and dataset CRA001160, comprising 24 tumor and 11 normal samples, was acquired from the Genome Sequence Archive (accessed May 24, 2024). In addition, we included dataset GSE205013, which comprises 27 PAC-related samples (accessed June 26, 2024). For our analysis, we selected data from 68 patients representing various tumor stages. We processed the sequencing data using Cell Ranger v7.0 and primarily analyzed the scRNA-seq counts matrices with the Python package Scanpy v1.9.1 (12). To ensure data integrity, we applied Scrublet (default settings) for doublet detection on all scRNA-seq data (13). Cells classified as putative doublets were entirely removed from subsequent analyses, thereby minimizing potential artifacts in downstream clustering and differential expression steps. Additionally, we collected bulk RNA data, complete with detailed clinical information, from PAC patients. This data, including associated clinical records, was sourced from The Cancer Genome Atlas (TCGA) (accessed May 23, 2024). The International Cancer Genome Consortium (ICGC) (https://dcc.icgc.org, accessed on October 20, 2024). GSE62452 were downloaded from the Gene Expression Omnibus (GEO) database (https://www.ncbi.nlm.nih.gov/gds, accessed on 26 October 2024). In our preprocessing steps, we removed samples not related to PAC and made necessary corrections to the normal samples before proceeding with our analysis (14). We utilized the R package limma v3.52 for analyzing the bulk RNA gene expression matrices (15).

### 2.2 scRNA-seq clustering, visualization and cell annotation

After sequencing data preprocessing, we excluded low-quality cells using the following filtering criteria. Specifically, cells were retained only if they (1) expressed between 300 and 8,000 genes, (2) had fewer than 20% of transcripts mapping to mitochondrial genes, and (3) had a unique molecular identifier (UMI) count above 1,000. Cells failing any of these thresholds were removed from subsequent analyses. The remaining cells were then normalized using *scanpy.pp.normalize_tot*al (target_sum=1e4) and log-transformed. Next, we identified the top 2,250 highly variable genes (HVGs) via *sc.pp.highly_variable_genes i*n Scanpy (n_top_genes=2250). A Principal Component Analysis (PCA) was performed on these HVGs (40 principal components), followed by Harmony (16) integration to mitigate potential batch effects. We then conducted unsupervised clustering with the Leiden algorithm (17) (resolution=0.1) and visualized the resulting clusters using Uniform Manifold Approximation and Projection (UMAP). Cluster annotation proceeded in two steps. First, we cross-referenced known marker genes from the literature to assign preliminary labels. Second, we applied the *sc.tl.rank_genes_groups* function (Wilcoxon rank-sum test) to identify differentially expressed genes in each cluster, refining cluster labels and further elucidating the biological context of the cellular landscape in PAC.

### 2.3 InferCNV analysis

To evaluate somatic large-scale chromosomal copy number variation (CNV) in each ductal cell, we utilized the Python package inferCNVpy (version 0.3.0). The necessary data inputs, including a raw counts matrix, an annotation file, and a gene/chromosome position file, were meticulously prepared in line with the specifications outlined in the inferCNVpy documentation (https://github.com/icbi-lab/infercnvpy). We designated B cells and T cells as the reference normal cells for this analysis, applying the default settings of the package. We then classified the ductal cells based on their CNV scores. Cells with a CNV score exceeding the median value were categorized as malignant ductal cells. In contrast, those with scores below the median were identified as non-malignant ductal cells. Further, to gain deeper insights into the heterogeneity within the malignant ductal cell population, we re-clustered these cells using the Leiden algorithm. This approach allowed us to discern potentially distinct subgroups within the malignant category, thereby contributing to a more nuanced understanding of tumor cell diversity.

### 2.4 GSEA and gene ontology enrichment analysis

To gain a deeper understanding of the functions and biological processes of the gene set, GSEA and GO Enrichment Analysis were conducted. These analyses were performed using the “clusterProfiler” package (18). We specifically focused on the GO biological process category to reveal the roles of the gene set in cellular biological functions and pathways.

### 2.5 Consensus non-negative matrix factorization and measuring correlation between gene expression programs and tumor stages

The consensus non-negative matrix factorization (NMF) was conducted by implementing custom modifications to the cNMF v1.1 Python package, as well as by referencing the workflow code written by William L. Hwang (11, 19). We utilized hypergeometric testing and Pearson correlation to analyze the relationship between each program and tumor staging.

### 2.6 Transcription factor regulon analysis

The SCENIC analysis was performed using pySCENIC (v1.1.3) with default parameters (20). In short, we randomly picked out 300–500 cells from each cell cluster to construct a new gene-cell matrix. Regions for transcription factors (TF) searching were restricted to 10 k distance centered the transcriptional start site or 500 bp upstream of the TSSs. We utilized the RcisTarget databases for Homo sapiens (hg38, refseq_r80, SCENIC+ gene-based) to perform TF motif enrichment. Specifically, cis-regulatory modules and TF–target relationships were identified by matching enriched motifs to known TF binding sites in these databases. Cluster-specific TFs were then defined as the top 10 or 15 TFs showing the largest decrease in fold change relative to all other clusters (Wilcoxon rank-sum test). Finally, we visualized the resulting TF networks to highlight key regulators within each cluster.

### 2.7 Cell–cell interaction analysis

We used cellphoneDB based on cellphoneDB database v.4.0.0 to infer cell–cell interactions of selected ligand–receptor pairs between different cell subpopulations (21). The potential interaction strength between two cell subpopulations was predicted based on the expression of ligand-receptor pairs. The enriched ligand-receptor interactions between two cell subpopulations were calculated based on a permutation test. We extracted significant ligand-receptor pairs with *P* value < 0.001.

### 2.8 Cell line culture, CTSV gene knockdown, and wound healing assay

Capan-2 and MIA PaCa-2 cell lines were purchased from the American Type Culture Collection (ATCC). Capan-2 cells were maintained in RPMI 1640 medium, and MIA PaCa-2 cells were maintained in DMEM medium. Both media were supplemented with 10% fetal bovine serum (Gibco BRL, USA) and 1% penicillin–streptomycin. Cultures were incubated at 37°C with 5% CO_2_ in a humidified atmosphere. Two distinct short-hairpin RNA (shRNA) sequences targeting CTSV (sh-CTSV-A: 5′-TCGTCCTTCCAGTTCTACAAA-3′ and sh-CTSV-B: 5′-GCAACACACAGAAGATTATAT-3′) were designed and cloned into the pLKO.1-TRC plasmid. Briefly, annealed oligonucleotides were ligated into the pLKO.1-TRC vector at the relevant restriction sites (*EcoRI/AgeI*). The recombinant plasmids were then transformed into Stbl3 competent *Escherichia coli* for amplification. Positive colonies were selected on LB agar plates containing 100 μg/mL ampicillin. Correct insert orientation and sequence were verified by restriction enzyme digestion and Sanger sequencing prior to viral packaging.

To produce lentiviral particles, 293T cells were seeded in 10 cm dishes and co-transfected with 10 μg of the pLKO.1-CTSV-shRNA plasmid, 7.5 μg of psPAX2 packaging plasmid, and 2.5 μg of pMD2.G envelope plasmid using Lipofectamine 3000 (Invitrogen) according to the manufacturer’s protocol. After 48 hours, the viral supernatant was collected, filtered through a 0.45 μm syringe filter. Target cells (Capan-2 and MIA PaCa-2) were plated in 6-well plates and infected by replacing their culture medium with viral supernatant in the presence of 5 μg/mL Polybrene. Twenty-four hours post-infection, the medium was changed, and cells were selected with 4 μg/mL puromycin for 5 days to establish stable knockdown lines.

For functional validation, stable CTSV-knockdown cells and corresponding control cells were seeded in 6-well plates and grown to 90–95% confluence. A straight “scratch” was then made through the monolayer using a sterile 200 μL pipette tip, followed by gently washing with PBS to remove debris. The culture medium was replaced with serum-reduced or serum-free medium to minimize cell proliferation effects. Images were captured at 0 and 48 hours post-scratch using an inverted phase-contrast microscope. The distance between the wound edges was measured, and relative wound closure (%) was calculated to assess cell migration capability.

### 2.9 Statistical analysis

R version 4.2.1 was used for statistical analysis. We utilized Wilcoxon test under the circumstance of non-normal data distribution. Kaplan-Meier curves with log-rank statistics were used to compare OS. *P* < 0.05 was considered statistically significant.

## 3 Results

### 3.1 scRNA-seq profiling of PAC samples

**Figure 1:**
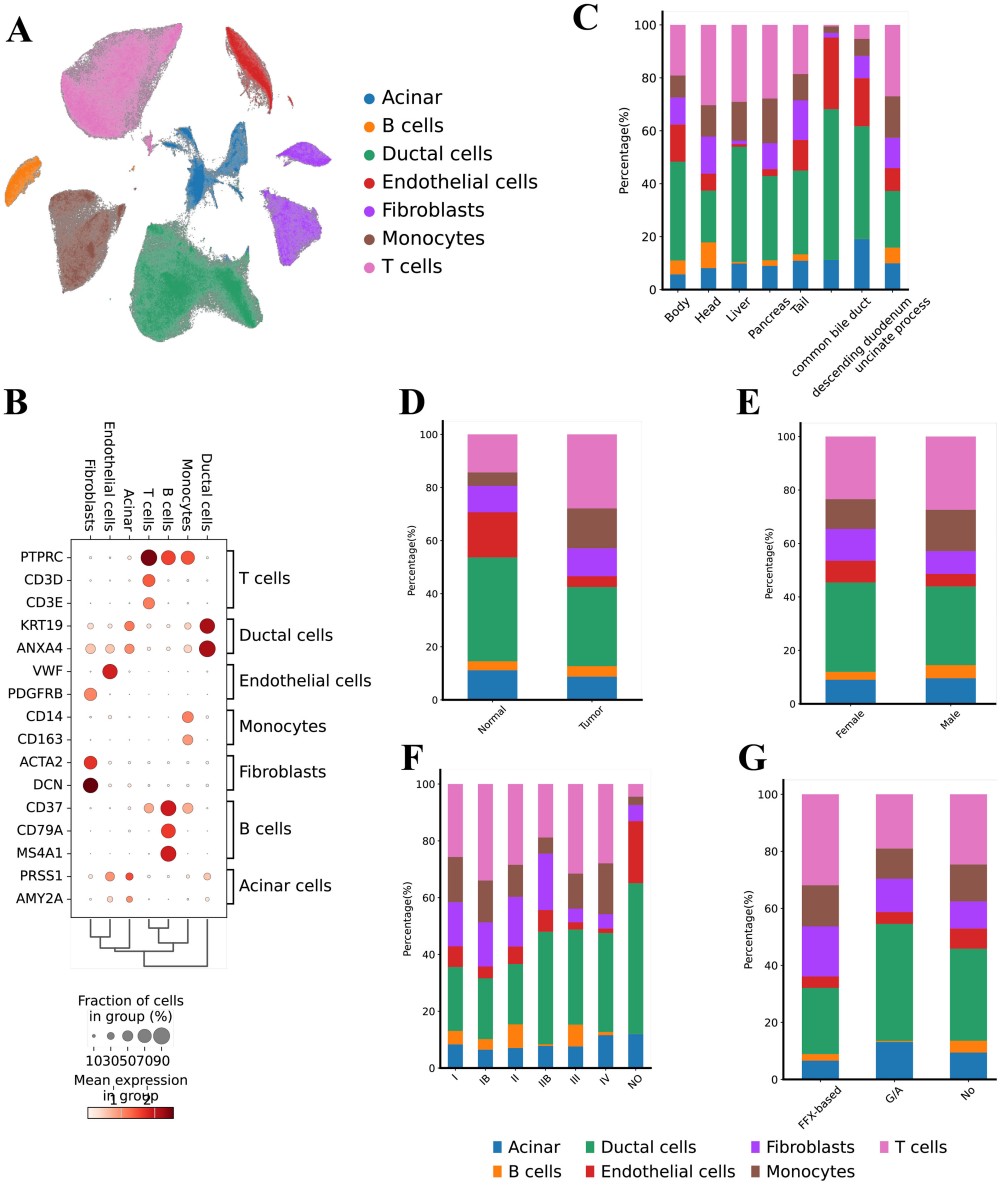
Single cell transcriptional landscape of PAC. **(A)** Uniform Manifold Approximation and Projection (UMAP) representation of the landscape of different PCA cell types, **(B)** Dot‐plots for the merged scRNA‐seq data demonstrates the marker expressions in the different cell types, **(C)** Bar chart illustrating the cell-type proportions across different anatomical locations (e.g., pancreatic head, body, liver metastasis), **(D)** Bar chart comparing cell-type distributions between tumor tissues and adjacent normal tissues, **(E)** Bar chart depicting the relative abundance of each cell type in male vs. female patients, **(F)** Bar chart showing cell-type composition stratified by clinical stages (I, II, III, and IV), **(G)** Bar chart presenting cell-type proportions under various treatment modalities (e.g., FOLFIRINOX, gemcitabine + nab-paclitaxel, untreated).

To systematically profile the transcriptional landscape of PAC, we collect scRNA-seq data from a total of 74 samples from 68 patients with PAC (**Figure 1**, **Supplementary Table S1**). These samples represented various clinical stages (I–IV), included both sexes, and covered multiple tumor locations. Following rigorous quality control, we retained a total of 506,736 high-quality cells for subsequent analyses. Unsupervised clustering analyses identified 7 major cell types (**Figure 1A**) including Ductal cells (KRT19, ANXA4), T cells (CD3D, CD3E), B cells (CD37, CD79A, MS4A1), Monocytes (CD14, CD163), Endothelial cells (VWF, PDGFRB), Acinar cells (PRSS1, AMY2A), and Fibroblasts (ACTA2, DCN) (**Figure 1B**).

Next, we analyzed the distribution of these cell types based on tumor location, tissue origin, gender, tumor stage, and treatment modality. In terms of tumor location, ductal cells were most prevalent in the common bile duct (57.00%), followed by endothelial cells (27.04%) (**Figure 1C**). The head and liver regions showed a higher proportion of T cells (30.33% and 29.08%, respectively). Regarding tissue origin, adjacent non-tumor tissue (Normal) had a higher proportion of endothelial cells (17.02%), whereas tumor tissues had higher proportions of monocytes (15.00%) and T cells (27.88%) (**Figure 1D**). We found no significant differences in cell type distribution based on gender (**Figure 1E**). From the perspective of tumor stages, T cells were most prevalent in stage Ib samples (34.00%) and least prevalent in normal samples (**Figure 1F**). Notably, acinar cells were elevated in samples without normal tissues and in stage IV samples (11.57% and 12.00%, respectively), while fibroblasts were more common in stages I and II (approximately 20%) but decreased in late-stage (IV) samples (around 5%). Analysis of treatment modalities revealed that samples treated with FFX had the highest proportion of T cells (31.89%), whereas samples treated with GA had the lowest proportion of B cells (**Figure 1G**).

In summary, our study comprehensively reveals the distribution characteristics of various cell types within the PAC TME and their differences across tumor location, tissue origin, tumor stage, and treatment modalities. Specifically, the significant increase in the proportion of ductal and endothelial cells within the bile duct suggests a critical role for these cells in PAC (**Supplementary Figure S1**). Additionally, the higher proportion of T cells in early-stage (Ib) and FFX-treated samples may reflect the importance of immune responses at different stages and under different treatment strategies. Furthermore, the increase in acinar cells in late-stage tumors and the higher proportion of fibroblasts in early-stage tumors provide important insights into the dynamic changes of cells during the progression of PAC.

### 3.2 Identification and characterization of malignant ductal cells

**Figure 2:**
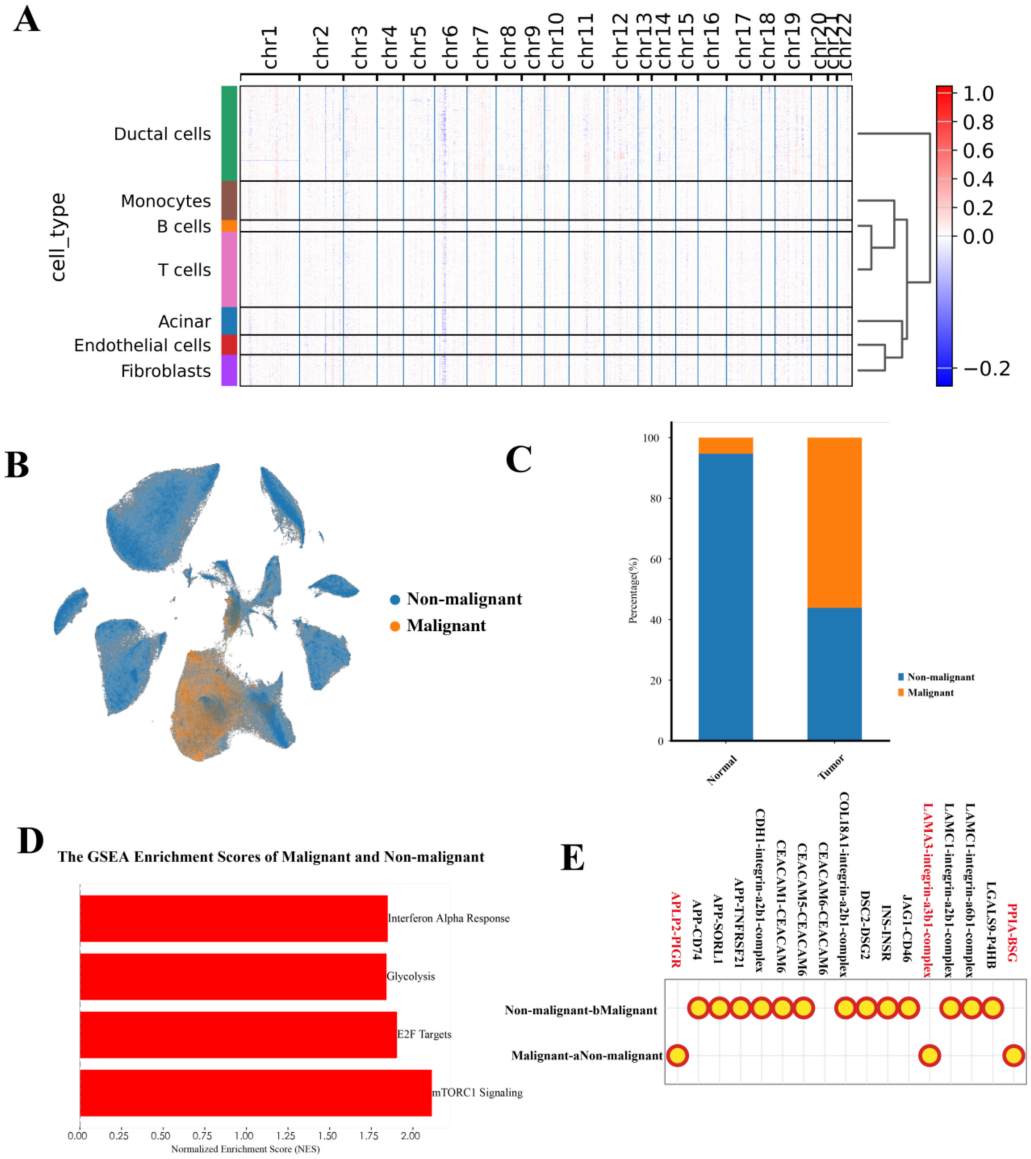
Characterization of malignant ductal cell states. **(A)** Heatmap of inferCNV analysis, illustrating large-scale chromosomal copy number variations in malignant versus non-malignant ductal cells. **(B)** UMAP plot showing the distribution of malignant and non-malignant ductal cells. **(C)** Bar chart depicting the distribution of malignant and non-malignant ductal cells across different tumor locations. **(D)** GSEA analysis of malignant and non-malignant ductal cells, highlighting significant pathways. **(E)** CellPhoneDB analysis of cell-cell interactions between malignant and non-malignant ductal cells.

To investigate the transcriptional heterogeneity in ductal cells, we distinguished 69,891 malignant ductal cells and 90,526 non-malignant ductal cells based on large-scale chromosomal CNVs (CNV score > 0.011) in each cell, inferred by inferCNVpy, from a total of 160,417 ductal cells (**Figures 2A, B**). To further validate our annotation of malignant ductal cells, we analyzed their distribution, finding that the malignant ductal cells were predominantly located in tumor regions (94.72%), supporting the accuracy of our identification (**Figure 2C**).

GSEA revealed significant activation of the mTORC1 signaling pathway, p53 signaling pathway, E2F targets, interferon alpha response, glycolysis pathway, and KRAS signaling pathway in malignant ductal cells (**Figure 2D**). Notably, the significant activation of the p53 signaling pathway, which differs from previous studies, suggests a unique heterogeneity within the PAC TME.

In the context of the TME, cell-cell interactions play a crucial role. We conducted an analysis of the interactions between malignant and non-malignant ductal cells, uncovering that malignant ductal cells engage in interactions through APLP2-PIGR, LAM3-integrin-a3b1-complex, and PPIA-BSG (**Figure 2E**). Taken together, these results confirm the presence of malignant ductal cells. Next, we sought to further dissect their heterogeneity and functional roles.

### 3.3 The heterogeneity of malignant ductal cells

**Figure 3:**
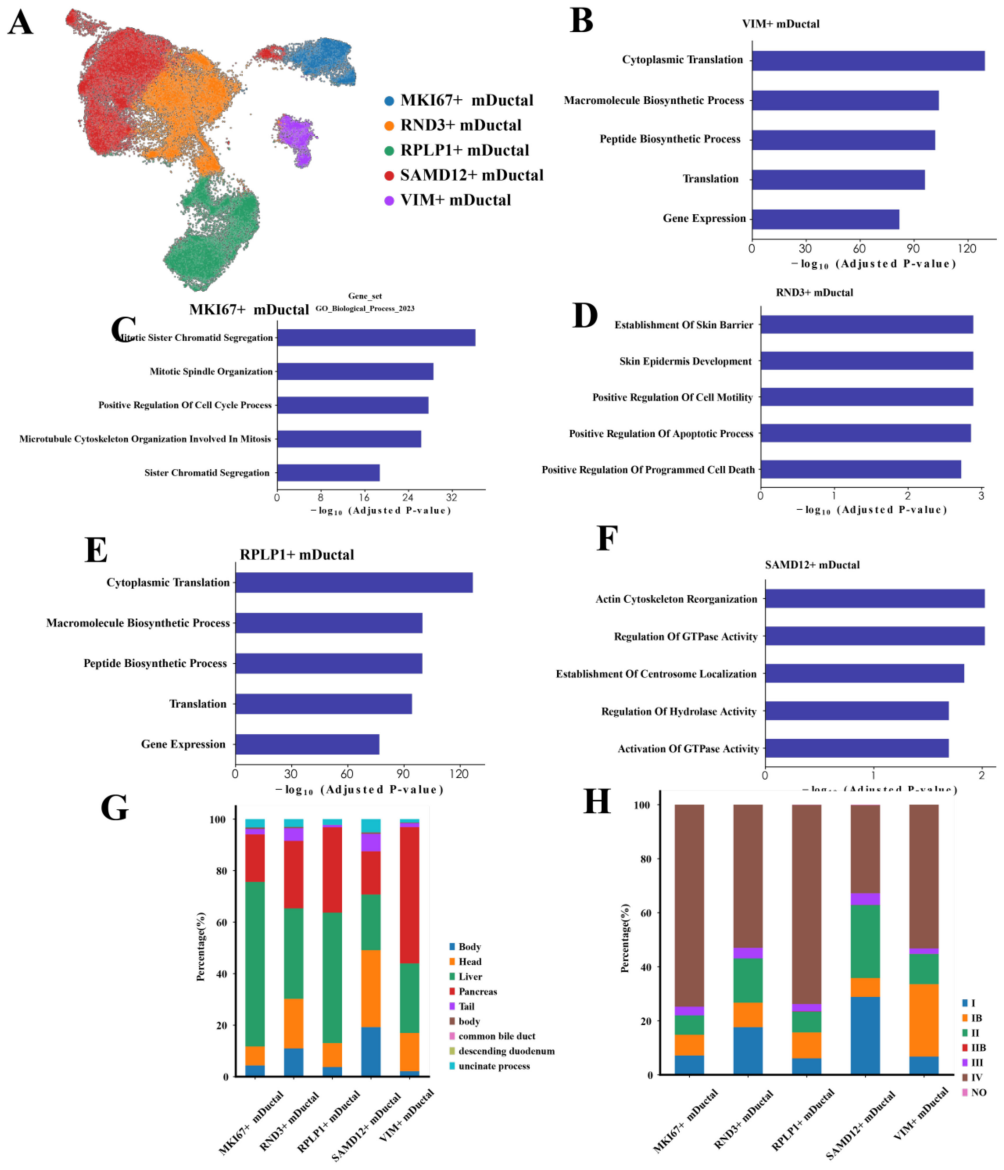
Characteristics of malignant ductal cells **(A)** UMAP plot showing the clustering of malignant ductal cells. **(B-F)** Bar charts illustrating the biological characteristics of each subpopulation: SAMD12+, RND3+, RPLP1+, MKI67+, and VIM+ ductal cells. **(G)** Distribution of different subpopulations across various tumor locations. **(H)** Distribution of different subpopulations across tumor stages.

We next focused on the malignant ductal cells. Through unsupervised clustering of 69,891 malignant ductal cells, we identified five distinct subpopulations (**Figure 3A**). Although we label these subpopulations by a single, highly upregulated marker gene—SAMD12, RND3, RPLP1, MKI67, and VIM—for convenience, the classification itself was based on the full set of differentially expressed genes identified via *sc.tl.rank_genes_groups* (Wilcoxon rank-sum test). Thus, each cluster is not exclusively defined by one marker but rather by a broader and unique transcriptional signature, underscoring that these subpopulations possess distinct gene expression profiles beyond any individual marker.

To characterize these subpopulations, we conducted GO enrichment analysis on the top 200 genes of the subpopulation cells. The results indicated that SAMD12+ cells were significantly enriched in processes related to actin cytoskeleton reorganization, regulation of GTPase activity, and centromere localization, suggesting these cells are in a state of dynamic remodeling, facilitating cell movement and structural reorganization (**Figure 3B**). RND3+ cells were significantly enriched in processes associated with skin barrier establishment, positive regulation of cell motility, and positive regulation of apoptosis, indicating roles in barrier function, motility regulation, and a higher propensity for apoptosis (**Figure 3C**). RPLP1+ cells showed significant enrichment in cytoplasmic translation, macromolecule biosynthesis, and gene expression, implying high levels of protein synthesis and metabolic activity (**Figure 3D**). MKI67+ cells were significantly enriched in mitosis, cell cycle regulation, and sister chromatid cohesion, highlighting their high proliferative state and active cell division (**Figure 3E**). VIM+ cells were significantly enriched in cytoplasmic translation and macromolecule biosynthesis, indicating robust protein synthesis and active metabolic processes (**Figure 3F**).

Next, we performed a detailed analysis of malignant ductal cells from different tumor locations, stages and gender. Our comparisons revealed significant biological differences among malignant ductal cells from various sites. Spatially, MKI67+ cells were predominantly located in the liver, suggesting a potential link to hepatic metastasis (**Figure 3G**). SAMD12+ cells were primarily found in the head of the pancreas, whereas VIM+ cells were widely distributed throughout the pancreas. The differential distribution of these genes across different locations may reflect distinct characteristics of PAC metastasis and local invasion. Regarding tumor stages, SAMD12+ cells were predominantly expressed in stage II tumors, VIM in stage Ib, and both MKI67 and RPLP1 exhibited higher expression levels in stage IV, indicating their roles in advanced tumor progression (**Figure 3H**). Notably, the high expression of MKI67+ cell is likely associated with the high proliferation rate and invasiveness of tumor cells. In the context of treatment modalities, due to the limited number of samples with treatment information, most samples were from the untreated group. However, existing data indicated that the proportion of SAMD12+ cells was lowest in the FFX-treated group, potentially reflecting the specific efficacy of this treatment on tumors located in the head of the pancreas. In terms of gender differences, we observed no significant variation in gene expression, suggesting that gender has minimal impact on the gene expression profiles of pancreatic ductal cells.

This suggests that the distinct distribution and associated biological processes of SAMD12+ and MKI67+ subpopulations across different stages and locations underscore their critical roles in PAC progression. Further investigation into these subpopulations may elucidate the molecular mechanisms underlying PAC.

### 3.4 NMF identified stage-related malignant processes

**Figure 4:**
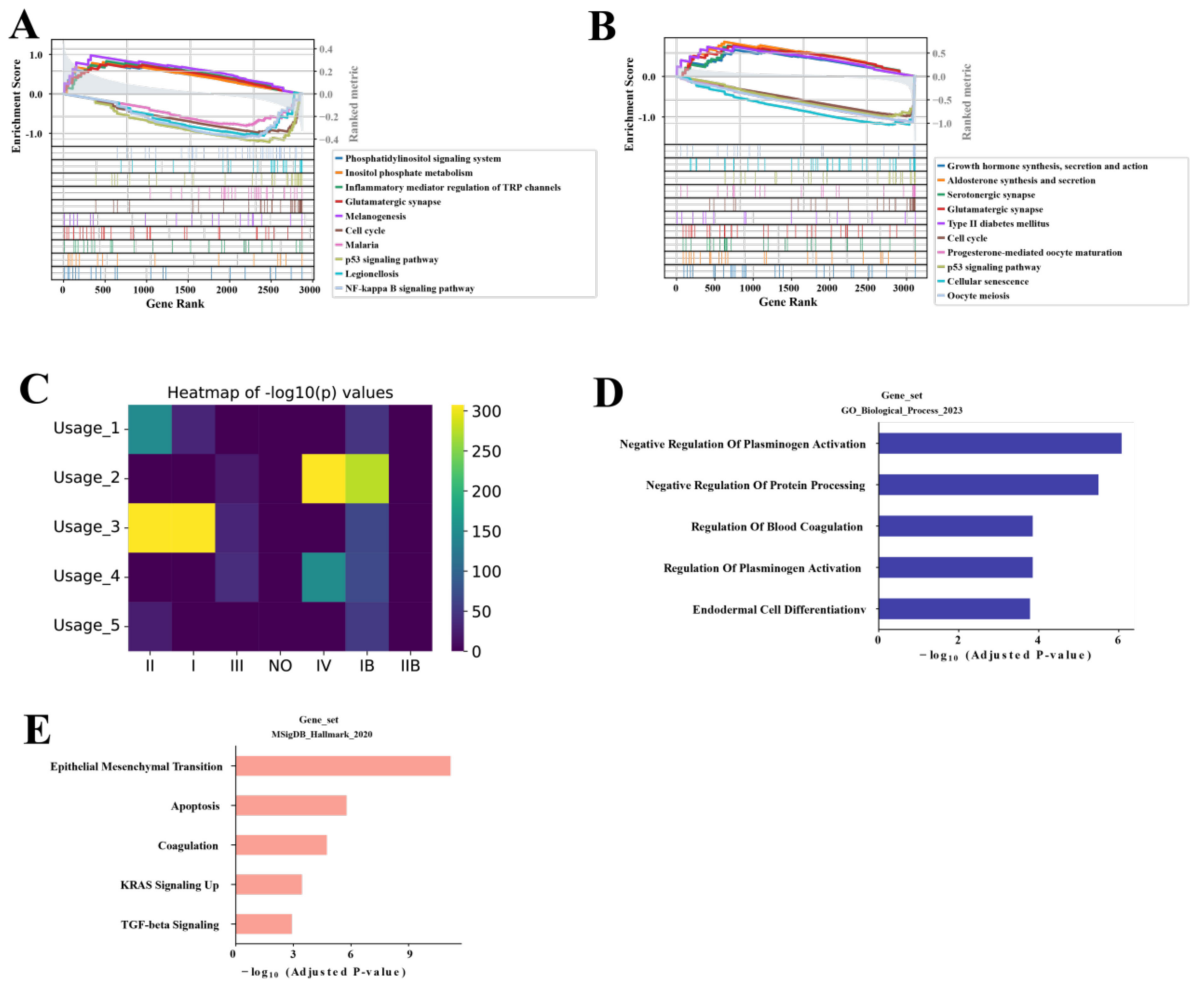
NMF analysis of malignancy-associated module in malignant ductal cells **(A, B)** GSEA of SAMD12+ and MKI67+ subpopulations, respectively. **(C)** Heatmap showing the correlation of five NMF modules with stages. **(D, E)** GO and MsigDB pathway analysis of the top 200 genes in the Usage_2 module, respectively.

To further explore the characteristics of the SAMD12+ and MKI67+ malignant ductal cell subpopulations, we conducted GSEA on these subpopulations, along with other relevant subpopulations. For the SAMD12 subpopulations, GSEA results indicated significant activation in pathways related to phosphatidylinositol signaling, inositol phosphate metabolism, TRP channels regulated by inflammatory mediators, glutamatergic synapses, and melanogenesis. Conversely, there was significant suppression in the NF-kappa B signaling pathway, cell cycle, malaria, p53 signaling pathway, and Legionellosis (**Figure 4A**). These results suggest that SAMD12-positive cells are in a state of high signal transduction and metabolic activity, with potentially lower proliferative and immune response capacities. This implies that SAMD12-positive cells might play critical roles in cell-cell communication and metabolic regulation while avoiding excessive cell proliferation and immune reactions.

For the MKI67 subpopulation, GSEA results showed significant activation in the cell cycle, progesterone-mediated oocyte maturation, p53 signaling pathway, cellular senescence, and oocyte meiosis. There was significant suppression in growth hormone synthesis, secretion and action, aldosterone synthesis and secretion, serotonergic synapse, glutamatergic synapse, and type II diabetes mellitus pathways (**Figure 4B**). These findings indicate that MKI67-positive cells are likely in a high proliferation and division state, with enhanced cell renewal and repair capabilities. This suggests that MKI67-positive cells may play crucial roles in tumor growth and progression, particularly in the rapid proliferation and division of tumor cells.

To further characterize the SAMD12 and MKI67 subpopulations, we performed NMF analysis and identified 5 distinct gene programs. From each program, we then extracted the top 200 genes and assessed their correlation with tumor stages (I–IV). Among these programs, Usage_2 showed the strongest association with advanced disease—specifically, stage IV (**Figure 4C**). We next performed GO and MsigDB enrichment analyses on the genes within Usage_2, revealing significant enrichment in pathways related to negative regulation of blood fibrinolysis, protein processing, blood coagulation, and endothelial cell differentiation (**Figure 4D**), as well as epithelial–mesenchymal transition, apoptosis, upregulated KRAS signaling, and TGF-beta signaling (**Figure 4E**). Taken together, these findings suggest that the Usage_2 module may play a key role in driving the malignancy of PAC cells, particularly in advanced stages.

### 3.5 Identification of prognostic genes associated with M0 macrophages from the Usage_2 module

**Figure 5:**
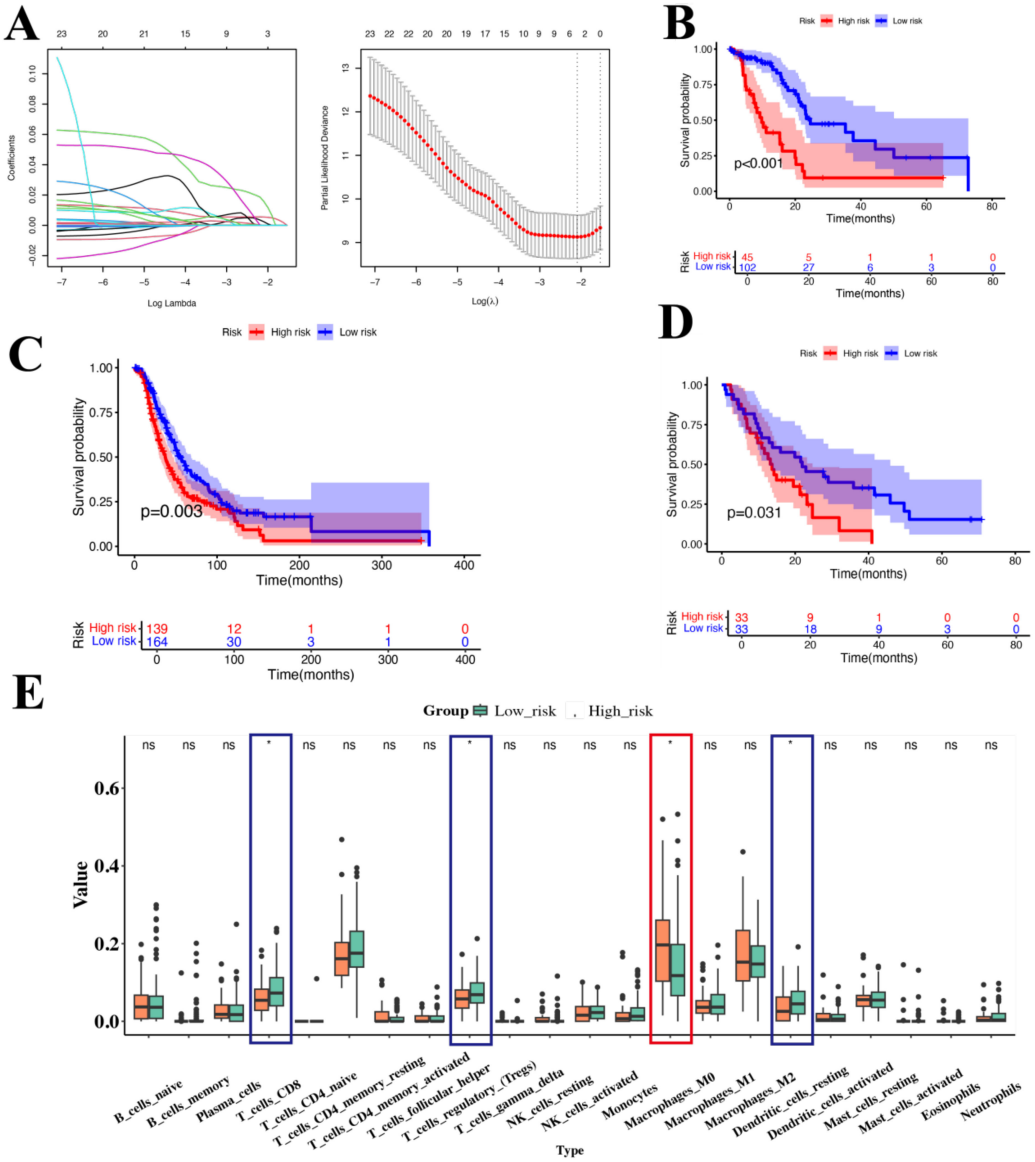
Prognostic analysis of genes associated with Usage_2 **(A)** Distribution of LASSO regression coefficients for survival associated AS events (left). Shrinkage parameter selection in the LASSO model using ten-fold cross-validation via minimum criteria (right). **(B)** KM survival curve of overall survival in TCGA-PAC. **(C)** KM survival curve of overall survival in ICGC. **(D)** KM survival curve of overall survival in GSE62452. **(E)** Box plot showed the ratio differentiation of 21 kinds of immune cells between TCGA-PAC samples with high-risk and low-risk groups. Significance levels were denoted as *P < 0.05. “ns” indicates no significant difference.

To evaluate the impact of these genes on PAC, we analyzed their relationship with prognosis using data from TCGA. First, univariate Cox regression analysis identified 23 genes significantly associated with prognosis of PAC (**Supplementary Table S2**). Then we applied Lasso regression and identify 3 best candidate genes (ANLN, NT5E, and CTSV) to generate risk scores (**Figure 5A**). The risk score formula was as follows: Risk score = (0.01487 × expression ANLN) + (0.00683 × expression NT5E) +(0.04551 × expression CTSV). Finally, we divided patients into high-risk and low-risk groups according to the median risk score for the TCGA-PAC data. KM analysis showed that the high-risk group had significantly worse prognosis compared with the low-risk group (*P* < 0.001, **Figure 5B**). Notably, the overall C-index of this LASSO-based model reached 0.69, indicating a moderate capacity to distinguish outcomes among TCGA-PAC patients.

We then validated these findings in two independent cohorts of PAC patients (ICGC and GSE62452 datasets), comprising a total of 369 samples. The results showed that the high-risk group had a poorer prognosis than those in the low-risk group (*P*=0.003, P < 0.031, respectively, **Figures 5C, D**). Considering the role of the TME, we used CIBERSORT to analyze immune cell infiltration in different groups. We found that M0 macrophages were significantly higher in the high-risk group, while CD8+ T cells and dendritic cells (DCs) were significantly lower. This suggests that these genes may be involved in macrophage differentiation, which in turn may be related to the progression of PAC. Collectively, these data indicated that ANLN, NT5E, and CTSV may play a pivotal role in PAC and are likely associated with M0 macrophages. Given that ANLN, NT5E, and CTSV are closely associated with M0 macrophage infiltration, we next examined how these high-expression malignant ductal cells interact with macrophages in the TME.

### 3.6 M0 macrophages interact with malignant ductal cells expressing high levels of ANLN, NT5E, and CTSV via CXCL14-CXCR4

To further understand the specific roles of the prognostic genes ANLN, NT5E, and CTSV, we analyzed their expression in scRNA data. We focused particularly on their relationship with M0 macrophages to reveal their potential functions within the TME.

**Figure 6:**
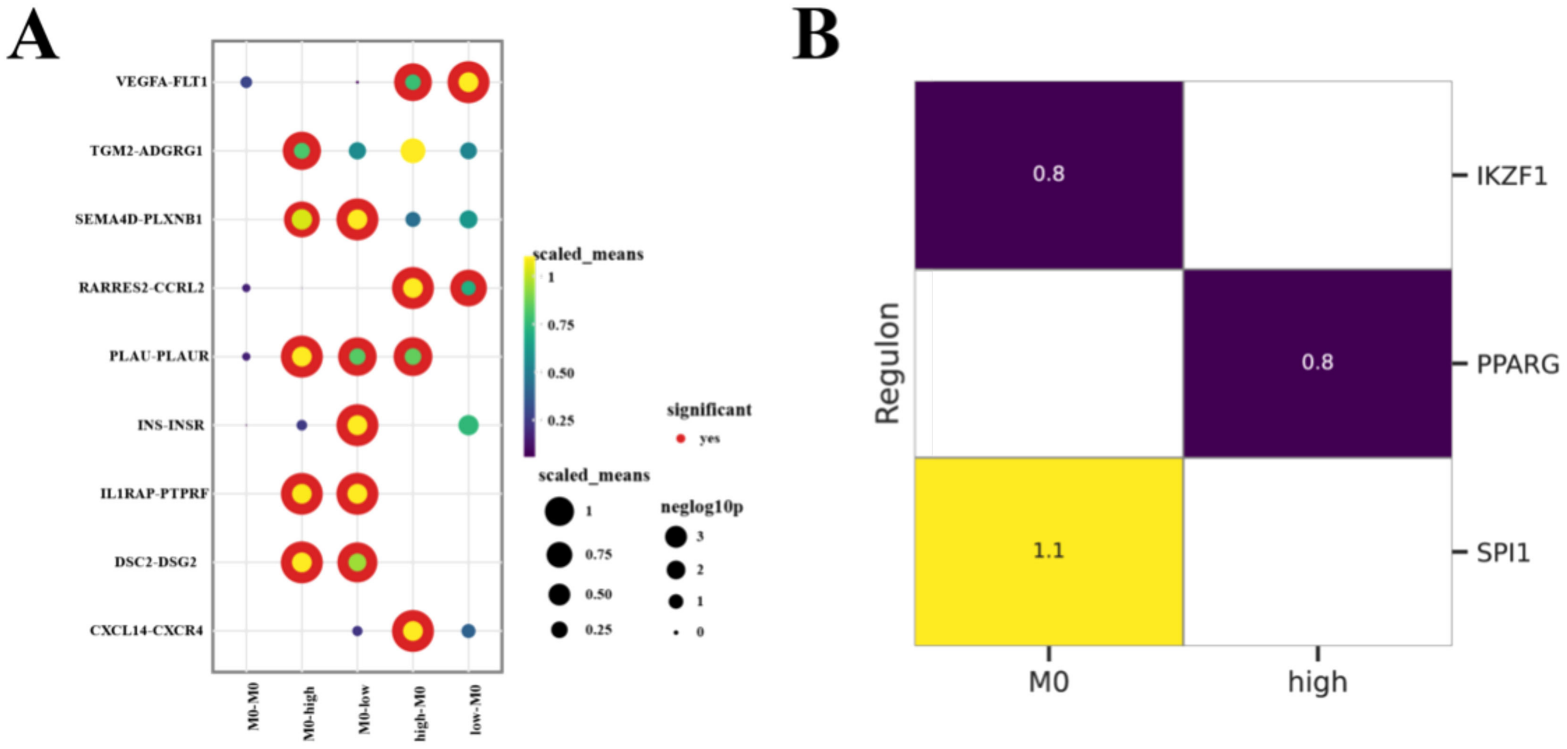
M0 Macrophages Interact with Malignant Ductal Cells **(A)** Bubble plots illustrate the intercellular interactions among M0 macrophages and Malignant Ductal Cells, with bubble size and color indicating the significance and strength of each ligand–receptor pair. **(B)** Heatmap comparing the most active TFs across different subpopulations, depicted as Z-scores.

First, we divided the malignant ductal cell subpopulations into high and low expression groups based on the expression levels of these three genes. In both the high and low expression groups, we performed cell interaction analysis of M0 macrophages to reveal how the expression of these genes affects the behavior and function of M0 macrophages. Using CellPhoneDB for intercellular communication analysis, we found that the interactions between M0 macrophages and ductal cells were significantly increased in the high expression group, suggesting that these genes play key roles in regulating the TME (**Figure 6A**).

Through Pyscenic analysis, we further explored the transcription factor networks of each subpopulation. In the high expression group, we found that PPARG was the main transcription factor, while SPI1 was the key transcription factor in M0 macrophages (**Figure 6B**). Combining the results of cell interaction analysis, we identified important ligand-receptor pairs between M0 macrophages and high expression group ductal cells: IL1RAP (ligand from M0 macrophages) - PTPRF (receptor on high expression group ductal cells) and CXCL14 (ligand from high expression group ductal cells) - CXCR4 (receptor on M0 macrophages). It is particularly noteworthy that the IL1RAP-PTPRF pair is not only present between M0 macrophages and high expression group ductal cells but also between M0 macrophages and low expression group ductal cells, suggesting that it may have different functions in ductal cells with varying malignancy levels. These findings indicated that the widespread presence of IL1RAP-PTPRF may indicate its general importance in the TME, while the specificity of CXCL14-CXCR4 interactions may be related to the highly malignant ductal cells.

### 3.7 Effect of CTSV knockdown on migration of PAC cells

**Figure 7:**
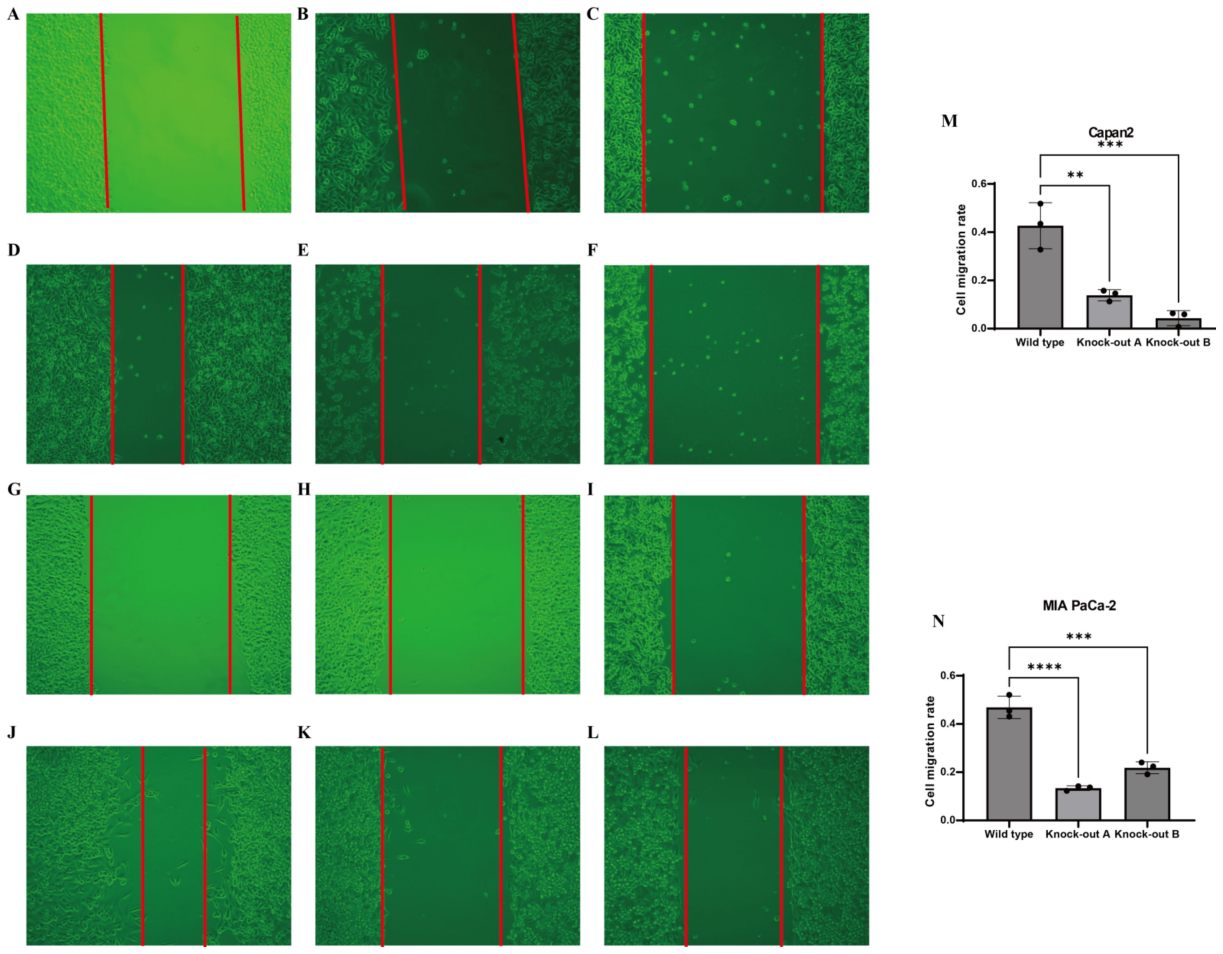
*In vitro* experimental validation. **(A-F)** Representative microscopic images showing the effects of gene knockdown in Capan-2 cells. **(A)** Control (0h), **(B)** shRNA targeting CTSV A (0h), **(C)** shRNA targeting CTSV B (0h), **(D)** Control (48h), **(E)** shRNA targeting CTSV A (48h), and **(F)** shRNA targeting CTSV B (48h). **(G-L)** Representative microscopic images showing the effects of gene knockdown in MIAPaCa cells. **(G)** Control (0h), **(H)** shRNA targeting CTSV A (0h), **(I)** shRNA targeting CTSV B (0h), **(J)** Control (48h), **(K)** shRNA targeting CTSV A (48h), and **(L)** shRNA targeting CTSV B (48h). **(M)** Invasion rate of Capan-2 cells following gene knockdown with different shRNAs, as measured by a transwell assay. **(N)** Invasion rate of MIAPaCa cells following gene knockdown with different shRNAs, as measured by a transwell assay. Data are presented as mean ± SD from three independent experiments. **P < 0.01, ***P < 0.001 and ***P < 0.0001.

CTSV has a significant effect on the proliferation and migration of PAC cell lines. In order to further investigate the functional significance of CTSV in PAC cell lines, we investigated the effects of CTSV knockdown on two specific cell lines, Capan2 and MIA paca2. The scratch repair experiment results showed that the cell migration rate of the CTSV knockdown group was significantly reduced compared to the control group (**Figures 7A–N**), indicating that CTSV knockdown significantly inhibited the proliferation and migration ability of PAC cells.

## 4 Discussion

PAC is a highly malignant and difficult-to-treat cancer with extremely high mortality and recurrence rates (1). Despite some progress in treatment methods, the prognosis for PAC remains poor due to its complex TME and highly heterogeneous cell composition. Therefore, understanding the cellular composition and interactions within the PAC TME is crucial for developing more effective therapeutic strategies. In this study, we comprehensively characterized the complex TME landscapes of PAC, demonstrating TME features and properties associated with various PAC clinical characteristics, including tumor location, tissue type, stage, and sex.

We found that fibroblasts are more abundant in the early stages of PAC, indicating their important role in the initial phases of PAC. Fibroblasts not only support tumor growth and metastasis but also influence the structure and function of the TME by secreting extracellular matrix (ECM) components and various growth factors (22–25). Additionally, we observed a higher abundance of T cells and monocytes in tumor tissues compared with normal controls, indicating an immune component in PAC progression. However, recent single-cell studies Zheng et al. (26). and Cheng et al. (27) have highlighted that many of these T cells may be in an exhausted state, while monocytes often develop into immunosuppressive macrophage subtypes in PAC, further exacerbating tumor progression Consistent with these reports, our findings suggest a significant immune infiltration, but do not fully resolve the activation status or phenotypic diversity (e.g., M1 vs. M2 macrophages, effector vs. exhausted T cells) within these populations. Future work could involve more detailed analyses of checkpoint markers (e.g., PD-1, TIM-3) in T cells and polarization markers (e.g., CD163, CD206) in macrophages to delineate their functional states and potential therapeutic implications. This underscores the complexity of the TME and the need for deeper mechanistic studies to clarify how immune cells can both restrain and promote tumor growth.

In the TME, cancer cells, stromal fibroblasts, endothelial cells, and infiltrating immune populations engage in a dynamic network of reciprocal signaling—via cytokines, chemokines, growth factors, receptor–ligand interactions, and extracellular vesicles—that orchestrates tumor progression, angiogenesis, and immune modulation (28). Cancer cells interact not only with immune cells but also affect the behavior of normal epithelial cells through cytokines and chemokines. Studies have shown that cancer cells can induce epithelial-mesenchymal transition in normal epithelial cells by secreting transforming growth factor-β and epidermal growth factor, increasing cell migration and invasion capabilities (29, 30). In our study, we found that malignant and non-malignant ductal cells can interact through APLP2-PIGR, LAM3-integrin-a3b1-complex, and PPIA-BSG. These interactions may contribute to the malignancy of normal ductal cells. APLP2-PIGR and LAM3-integrin-a3b1-complex interactions aid in cell adhesion and signal transduction, which may play critical roles in tumor progression (31, 32). PPIA-BSG interactions are related to the proliferation and invasiveness of tumor cells (33, 34).

GSEA analysis revealed that malignant ductal cells are in a highly metabolic state, indicating a high energy demand to support their rapid proliferation and growth. Notably, our study also found that the p53 pathway is significantly activated, further illustrating the heterogeneity of the PAC TME. The activation of the p53 signaling pathway in PAC may be associated with stress responses and DNA damage repair mechanisms in tumor cells (30). Moreover, the activation of the p53 pathway might be related to metabolic reprogramming in tumor cells, providing the necessary energy and metabolic intermediates to support their growth (35).

The heterogeneity of the TME is a key focus of research for identifying new therapeutic directions. Among the malignant ductal subpopulations we identified, SAMD12+ and MKI67+ exhibited distinct gene expression patterns suggestive of higher proliferative and metabolic activity relative to other clusters (**Figures 3C, F**). In particular, GSEA revealed that SAMD12+ cells may upregulate pathways involved in phosphatidylinositol signaling and inositol phosphate metabolism. Although these results imply a heightened metabolic and signaling state, further functional validation is necessary to confirm these observations (36, 37).

Through NMF analysis, we identified the Usage_2 module related to malignancy progression and integrated it with TCGA data, determining three prognostic genes: ANLN, NT5E, and CTSV. These genes were validated by two independent datasets, further supporting their significant roles in PAC.

ANLN (Anillin) is a cell division-related protein that plays a critical role in mitosis and cytokinesis (38). High ANLN expression is typically associated with tumor invasiveness and poor prognosis in various cancers (38–41). For instance, high ANLN expression correlates with enhanced proliferation and migration capabilities of tumor cells in lung adenocarcinoma and breast cancer (40, 41). In PAC, ANLN overexpression may promote rapid division and proliferation of tumor cells, accelerating tumor progression.

NT5E encodes CD73, a cell surface enzyme involved in adenosine production. Adenosine exerts immunosuppressive effects in the TME, inhibiting the activity of T cells and natural killer cells, thereby promoting tumor immune evasion (42). High CD73 expression is associated with poor prognosis and tumor resistance in several cancers, including breast and colorectal cancer (43–45). In PAC, high NT5E expression might suppress antitumor immune responses via the adenosine signaling pathway, promoting tumor growth and metastasis.

CTSV (Cathepsin V) is a lysosomal cysteine protease involved in protein degradation and remodeling (46). High CTSV expression is associated with enhanced invasion and metastasis of tumor cells in various cancers, such as breast and liver cancer (47, 48). In PAC, CTSV may enhance tumor cell migration and invasion by promoting extracellular matrix degradation and remodeling.

Immune infiltration analysis revealed that M0 macrophages are significantly more abundant in the high-risk group defined by these three genes, suggesting that they play a crucial role in PAC progression. M0 macrophages are regarded as un-activated or initial-state macrophages that can be polarized into various functional states (e.g., M1 or M2) within the TME (49, 50). Our ScRNA data further indicate that malignant ductal cells in the high-expression group of these three genes may interact with M0 macrophages through the CXCL14–CXCR4 axis, potentially facilitating an immunosuppressive environment.

Myeloid plasticity is increasingly recognized as an important therapeutic target in PAC. For instance, Zhou et al. (51) demonstrated that blocking the CD47/SIRPα axis can reprogram macrophages to a more phagocytic, antitumor phenotype, thereby overcoming immune evasion. In light of our findings that M0 macrophages accumulate in high-risk PAC subgroups and may skew toward a protumoral (M2-like) phenotype, targeting macrophage polarization—whether via CD47/SIRPα blockade or other agents—could be a promising strategy. Future studies are warranted to determine whether combining such macrophage-focused therapies with established treatments might improve patient outcomes in PAC”.

In our study, the CXCL14–CXCR4 interaction emerged as a particularly notable pathway between malignant ductal cells and M0 macrophages. CXCL14 is a chemokine whose function can be highly context-dependent, capable of promoting fibroblast activation and tumor progression or, alternatively, recruiting NK or T cells in a more antitumor capacity (52),CXCR4, its corresponding receptor, is widely expressed on various immune cells and tumor cells. Our data suggest that, in the PAC context we studied, CXCL14-expressing malignant ductal cells may help establish immunosuppressive interactions with M0 macrophages, potentially contributing to immune evasion or tumor progression. Nevertheless, these observations remain correlative, and further functional assays (e.g., CXCL14/CXCR4 blockade or knockdown) are needed to confirm this mechanism and clarify whether antitumoral pathways might also be engaged under certain TME conditions. Such complexity highlights the importance of analyzing not just ligand–receptor pairings, but the broader immune context in which they operate.

Moreover, we found that M0 macrophages interact with both high- and low- malignant ductal cells via the IL1RAP–PTPRF axis. This widespread presence suggests that IL1RAP–PTPRF may function as a more general communication pathway within the TME, rather than being confined to late-stage or highly aggressive cells. IL1RAP, an interleukin-1 receptor accessory protein, associates with PTPRF (protein tyrosine phosphatase receptor type F) to modulate diverse signaling pathways, including inflammation and cell proliferation (53). In PAC, our data indicate that such signals might regulate the survival and proliferation of ductal cells across different malignancy states, ultimately impacting overall tumor progression.

Beyond basic cell growth, it is plausible that IL1RAP–PTPRF contributes to cellular plasticity—for instance, influencing how malignant cells transition between less- and more-aggressive phenotypes—or to immune tolerance, by shaping how ductal cells interact with macrophages and other immune constituents in the TME (54, 55). Although our single-cell analysis highlights the correlative nature of these interactions, further mechanistic studies (e.g., IL1RAP or PTPRF knockdown, co-culture experiments with macrophages) are needed to verify whether perturbing this axis could shift ductal cell behavior or macrophage polarization. Such investigations would clarify whether IL1RAP–PTPRF indeed serves as a unifying link between tumor aggressiveness and immunosuppression, thereby offering a potential avenue for therapeutic intervention.

Pyscenic analysis showed that SPI1 is the transcription factor for IL1RAP, further supporting the critical role of M0 macrophages in regulating IL1RAP expression (56). SPI1 is a key transcription factor essential for the development and function of myeloid cells (57). The regulation of IL1RAP by SPI1 indicates that M0 macrophages might modulate their behavior in the TME through SPI1-mediated pathways, influencing the characteristics of malignant ductal cells.

## 5 Conclusions

In this study, we integrated multiple single-cell datasets to identify two key subpopulations of highly malignant ductal cells and three prognostic genes (ANLN, NT5E, CTSV) in PAC, then confirmed these genetic findings using both TCGA and independent external cohorts. Our analysis also revealed that M0 macrophages and malignant ductal cells likely communicate through the IL1RAP–PTPRF and CXCL14–CXCR4 axes, hinting at a dynamic interplay that may foster an immunosuppressive TME. Meanwhile, functional assays showed that knocking down CTSV significantly impaired PAC cell migration and proliferation, underscoring the relevance of these molecular discoveries in disease progression. Despite these promising insights, several limitations warrant discussion. First, the bulk of our conclusions about macrophage–ductal cell cross-talk relies on computational analyses (CellPhoneDB and SCENIC), without *in vivo* corroboration. Further mechanistic studies—such as co-culturing ductal cells with primary macrophages or employing genetically engineered mouse models—are needed to confirm the physiological roles of these ligand–receptor interactions. Second, although we observed more M0 macrophages in the high-risk group, we did not dissect specific immune phenotypes (e.g., M1 vs. M2) in depth, which could clarify how macrophage plasticity shapes the local TME. Lastly, while we validated CTSV’s effect *in vitro*, other putative drivers identified here (ANLN, NT5E) remain to be explored functionally. Addressing these gaps in future work may illuminate new therapeutic angles—especially interventions aimed at modulating macrophage polarization or blocking key ligand–receptor pathways in advanced PAC. In sum, our findings offer an integrative single-cell perspective on malignant ductal cell subpopulations and immune components in PAC, laying a foundation for targeted interventions aimed at blocking pro-tumoral crosstalk and improving patient outcomes.

## Data Availability

The original contributions presented in the study are included in the article/**Supplementary Material**. Further inquiries can be directed to the corresponding author.

## Supplementary material

Supplementary Table 1 The clinical information of samples included in the present study.

## References

[1] Siegel RL Kratzer TB Giaquinto AN Sung H Jemal A. Cancer statistics, 2025. Ca-a Cancer J Clin. (2025) 75:10–45. doi: 10.3322/caac.21871 PMC11745215 39817679

[2] Ramai D Smith ER Wang Y Huang Y Obaitan I Chandan S. Epidemiology and socioeconomic impact of pancreatic cancer: an analysis of the global burden of disease study 1990-2019. Digest Dis Sci. (2024) 69:1135–42. doi: 10.1007/s10620-024-08292-1 38383939

[3] Jiang Z Zheng X Li M Liu M. Improving the prognosis of pancreatic cancer: insights from epidemiology, genomic alterations, and therapeutic challenges. Front Med. (2023) 17:1135–69. doi: 10.1007/s11684-023-1050-6 38151666

[4] Zemek RM Anagnostou V Pires da Silva I Long GV Lesterhuis WJ. Exploiting temporal aspects of cancer immunotherapy. Nat Rev Cancer. (2024) 24:480–97. doi: 10.1038/s41568-024-00699-2 38886574

[5] Bear AS Vonderheide RH O’Hara MH. Challenges and opportunities for pancreatic cancer immunotherapy. Cancer Cell. (2020) 38:788–802. doi: 10.1016/j.ccell.2020.08.004 32946773 PMC7738380

[6] Paredes-Moscosso SR Nathwani AC. 10 years of BiTE immunotherapy: an overview with a focus on pancreatic cancer. Front Oncol. (2024) 14:1429330. doi: 10.3389/fonc.2024.1429330 PMC11696039 39759138

[7] Stuart T Satija R. Integrative single-cell analysis. Nat Rev Genet. (2019) 20:257–72. doi: 10.1038/s41576-019-0093-7 30696980

[8] Yu W Biyik-Sit R Uzun Y Chen C-H Thadi A Sussman JH. Longitudinal single-cell multiomic atlas of high-risk neuroblastoma reveals chemotherapy-induced tumor microenvironment rewiring. Nat Genet. (2025) 1–13. doi: 10.1038/s41588-025-02158-6 PMC12081299 40229600

[9] Elyada E Bolisetty M Laise P Flynn WF Courtois ET Burkhart RA. Cross-Species single-Cell analysis of pancreatic ductal adenocarcinoma reveals antigen-Presenting cancer-Associated fibroblasts. Cancer Discov. (2019) 9:1102–23. doi: 10.1158/2159-8290.CD-19-0094 PMC6727976 31197017

[10] Peng J Sun B-F Chen C-Y Zhou J-Y Chen Y-S Chen H. Single-cell RNA-seq highlights intra-tumoral heterogeneity and Malignant progression in pancreatic ductal adenocarcinoma. Cell Res. (2019) 29:725–38. doi: 10.1038/s41422-019-0195-y PMC6796938 31273297

[11] Hwang WL Jagadeesh KA Guo JA Hoffman HI Yadollahpour P Reeves JW. Single-nucleus and spatial transcriptome profiling of pancreatic cancer identifies multicellular dynamics associated with neoadjuvant treatment. Nat Genet. (2022) 54:1178–+. doi: 10.1038/s41588-022-01134-8 PMC10290535 35902743

[12] Wolf FA Angerer P Theis FJ. SCANPY: large-scale single-cell gene expression data analysis. Genome Biol. (2018) 19:1–5. doi: 10.1186/s13059-017-1382-0 PMC5802054 29409532

[13] Wolock SL Lopez R Klein AM. Scrublet: computational identification of cell doublets in single-cell transcriptomic data. Cell Syst. (2019) 8:281–+. doi: 10.1016/j.cels.2018.11.005 PMC6625319 30954476

[14] PeranIMadhavanSByersSWMcCoyMD. Curation of the pancreatic ductal adenocarcinoma subset of the cancer genome atlas is essential for accurate conclusions about survival-related molecular mechanisms. Clin Cancer Res. (2018) 24:3813–9. doi: 10.1158/1078-0432.CCR-18-0290 29739787

[15] Ritchie ME Phipson B Wu D Hu Y Law CW Shi W. *limma* powers differential expression analyses for RNA-sequencing and microarray studies. Nucleic Acids Res. (2015) 43(7):e47–e47. doi: 10.1093/nar/gkv007 25605792 PMC4402510

[16] Korsunsky I Millard N Fan J Slowikowski K Zhang F Wei K. Fast, sensitive and accurate integration of single-cell data with Harmony. Nat Methods. (2019) 16(12):1289–96. doi: 10.1038/s41592-019-0619-0 PMC6884693 31740819

[17] Traag VA Waltman L van Eck NJ. From Louvain to Leiden: guaranteeing well-connected communities. Sci Rep. (2019) 9(1):1–12. doi: 10.1038/s41598-019-41695-z PMC6435756 30914743

[18] Wu TZ Hu EQ Xu SB Chen MJ Guo PF Dai ZH. clusterProfiler 4.0: A universal enrichment tool for interpreting omics data. Innovation. (2021) 2(3). doi: 10.1016/j.xinn.2021.100141 PMC8454663 34557778

[19] Kotliar D Veres A Nagy MA Tabrizi S Hodis E Melton DA. Identifying gene expression programs of cell-type identity and cellular activity with single-cell RNA-Seq. Elife. (2019) 8:e43803. doi: 10.7554/eLife.43803 31282856 PMC6639075

[20] Aibar S González-Blas CB Moerman T Van AHT Imrichova H Hulselmans G. SCENIC: single-cell regulatory network inference and clustering. Nat Methods. (2017) 14:1083–+. doi: 10.1038/nmeth.4463 PMC5937676 28991892

[21] Efremova M Vento-Tormo M Teichmann SA Vento-Tormo R. CellPhoneDB: inferring cell-cell communication from combined expression of multi-subunit ligand-receptor complexes. Nat Protoc. (2020) 15:1484–506. doi: 10.1038/s41596-020-0292-x 32103204

[22] Akanda MR Lubaba U Rahman MK Islam A Akter M Islam MS. Mechanistic role of stromal cancer-associated fibroblasts in tumorigenesis and brain metastasis: Highlighting drug resistance and targeted therapy. Pathol Res Pract. (2025) 269:155918. doi: 10.1016/j.prp.2025.155918 40120401

[23] Zhao J Lin E Bai Z Jia Y Wang B Dai Y. Cancer-associated fibroblasts induce sorafenib resistance of hepatocellular carcinoma cells through CXCL12/FOLR1 (vol 23, 1198, 2023). BMC Cancer. (2025) 25(1):1198. doi: 10.1186/s12885-025-13807-8 PMC10701976 38057830

[24] Takahashi K Shoda K Takiguchi K Higuchi Y Matsuoka K Nakayama T. Prognostic impact of stromal profiles educated by gastric cancer. Ann Surg Oncol. (2024) 31:2309–18. doi: 10.1245/s10434-023-14522-z 37919449

[25] Ozmen E Demir TD Ozcan G. Cancer-associated fibroblasts: protagonists of the tumor microenvironment in gastric cancer. Front Mol Biosci. (2024) 11. doi: 10.3389/fmolb.2024.1340124 PMC10982390 38562556

[26] Zheng L Qin S Si W Wang A Xing B Gao R. Pan-cancer single cell landscape of tumor-infiltrating T cells. Science. (2021) 374:1462–+. doi: 10.1126/science.abe6474 34914499

[27] Cheng S Li Z Gao R Xing B Gao Y Yang Y. A pan-cancer single-cell transcriptional atlas of tumor infiltrating myeloid cells. Cell. (2021) 184:792–+. doi: 10.1016/j.cell.2021.01.010 33545035

[28] Pradhan R Kundu A Kundu CN. The cytokines in tumor microenvironment: from cancer initiation-elongation-progression to metastatic outgrowth. Crit Rev Oncol Hematol. (2024) 196:104311. doi: 10.1016/j.critrevonc.2024.104311 38442808

[29] Hao Y Baker D ten Dijke P. TGF–mediated epithelial-mesenchymal transition and cancer metastasis. Int J Mol Sci. (2019) 20(11):2767. doi: 10.3390/ijms20112767 31195692 PMC6600375

[30] Janus P Kus P Jaksik R Vydra N Toma-Jonik A Gramatyka M. Transcriptional responses to direct and indirect TGFB1 stimulation in cancerous and noncancerous mammary epithelial cells. Cell Commun Signaling. (2024) 22(1):522. doi: 10.1186/s12964-024-01821-5 PMC11514872 39468555

[31] Asanprakit W Lobo DN Eremin O Bennett AJ. Expression of polymeric immunoglobulin receptor (PIGR) and the effect of PIGR overexpression on breast cancer cells. Sci Rep. (2023) 13(1):16606. doi: 10.1038/s41598-023-43946-6 37789066 PMC10547702

[32] Tsuruta D Kobayashi H Imanishi H Sugawara K Ishii M Jones JCR. Laminin-332-integrin interaction: A target for cancer therapy? Curr Med Chem. (2008) 15:1968–75. doi: 10.2174/092986708785132834 PMC2992754 18691052

[33] Wang S Li M Xing L Yu J. High expression level of peptidylprolyl isomerase A is correlated with poor prognosis of liver hepatocellular carcinoma. Oncol Lett. (2019) 18:4691–702. doi: 10.3892/ol.2019.10846 PMC6781733 31611978

[34] Han JM Jung HJ. Cyclophilin A/CD147 interaction: A promising target for anticancer therapy. Int J Mol Sci. (2022) 23(16):9341. doi: 10.3390/ijms23169341 36012604 PMC9408992

[35] Liu Y Su Z Tavana O Gu W. Understanding the complexity of p53 in a new era of tumor suppression. Cancer Cell. (2024) 42:946–67. doi: 10.1016/j.ccell.2024.04.009 PMC11190820 38729160

[36] Connor AA Gallinger S. Pancreatic cancer evolution and heterogeneity: integrating omics and clinical data. Nat Rev Cancer. (2022) 22:131–42. doi: 10.1038/s41568-021-00418-1 34789870

[37] El Kaoutari A Fraunhoffer NA Hoare O Teyssedou C Soubeyran P Gayet O. Metabolomic profiling of pancreatic adenocarcinoma reveals key features driving clinical outcome and drug resistance. Ebiomedicine. (2021) 66. doi: 10.1016/j.ebiom.2021.103332 PMC8054161 33862584

[38] Cao Y-F Xie L Tong B-B Chu M-Y Shi W-Q Li X. Targeting USP10 induces degradation of oncogenic ANLN in esophageal squamous cell carcinoma. Cell Death Differ. (2023) 30:527–43. doi: 10.1038/s41418-022-01104-x PMC9950447 36526897

[39] Cui Z Mo J Song P Wang L Wang R Cheng F. Comprehensive bioinformatics analysis reveals the prognostic value, predictive value, and immunological roles of ANLN in human cancers. Front Genet. (2022) 13. doi: 10.3389/fgene.2022.1000339 PMC9527346 36199577

[40] Xu J Zheng H Yuan S Zhou B Zhao W Pan Y. Overexpression of ANLN in lung adenocarcinoma is associated with metastasis. Thorac Cancer. (2019) 10:1702–9. doi: 10.1111/1759-7714.13135 PMC6669805 31268619

[41] Magnusson K Gremel G Ryden L Ponten V Uhlen M Dimberg A. ANLN is a prognostic biomarker independent of Ki-67 and essential for cell cycle progression in primary breast cancer. BMC Cancer. (2016) 16:1–13. doi: 10.1186/s12885-016-2923-8 PMC5116155 27863473

[42] Xia C Yin S To KKW Fu L. CD39/CD73/A2AR pathway and cancer immunotherapy. Mol Cancer. (2023) 22(1):44. doi: 10.1186/s12943-023-01733-x 36859386 PMC9979453

[43] An R Wu C Tang C Zhang C Han F Xu Z. Blockade of CD73 potentiates radiotherapy antitumor immunity and abscopal effects via STING pathway. Cell Death Discov. (2024) 10(1):404. doi: 10.1038/s41420-024-02171-4 39285178 PMC11405876

[44] Bach N Winzer R Tolosa E Fiedler W Brauneck F. The clinical significance of CD73 in cancer. Int J Mol Sci. (2023) 24(14):11759. doi: 10.3390/ijms241411759 37511518 PMC10380759

[45] Lian W Jiang D Lin W Jiang M Zhang Y Wang H. Dual role of CD73 as a signaling molecule and adenosine-generating enzyme in colorectal cancer progression and immune evasion. Int J Biol Sci. (2024) 20:137–51. doi: 10.7150/ijbs.87440 PMC10750288 38164172

[46] Lecaille F Chazeirat T Saidi A Lalmanach G Cathepsin V. Molecular characteristics and significance in health and disease. Mol Aspects Med. (2022) 88(1): 01086. doi: 10.1016/j.mam.2022.101086 35305807

[47] Liu J Zhang W Wang Z Wang Y Li T Wang Y. Cathepsin V is correlated with the prognosis and tumor microenvironment in liver cancer. Mol Carcinogene. (2024) 63:400–16. doi: 10.1002/mc.23660 38051285

[48] Sereesongsaeng N McDowell SH Burrows JF Scott CJ Burden RE. Cathepsin V suppresses GATA3 protein expression in luminal A breast cancer. Breast Cancer Res. (2020) 22:1–12. doi: 10.1186/s13058-020-01376-6 PMC7726886 33298139

[49] Shapouri-Moghaddam A Mohammadian S Vazini H Taghadosi M Esmaeili S-A Mardani F. Macrophage plasticity, polarization, and function in health and disease. J Cell Physiol. (2018) 233:6425–40. doi: 10.1002/jcp.v233.9 PMC13482560 29319160

[50] Wang S Wang J Chen Z Luo J Guo W Sun L. Targeting M2-like tumor-associated macrophages is a potential therapeutic approach to overcome antitumor drug resistance. NPJ Precis Oncol. (2024) 8(1):31. doi: 10.1038/s41698-024-00522-z 38341519 PMC10858952

[51] Zhou Z Chen M-JM Luo Y Mojumdar K Peng X Chen H. Tumor-intrinsic SIRPA promotes sensitivity to checkpoint inhibition immunotherapy in melanoma. Cancer Cell. (2022) 40:1324–+. doi: 10.1016/j.ccell.2022.10.012 PMC9669221 36332624

[52] Augsten M Sjoberg E Frings O Vorrink SU Frijhoff J Olsson E. Cancer-associated fibroblasts expressing CXCL14 rely upon NOS1-derived nitric oxide signaling for their tumor-supporting properties. Cancer Res. (2014) 74:2999–3010. doi: 10.1158/0008-5472.CAN-13-2740 24710408

[53] Frenay J Bellaye P-S Oudot A Helbling A Petitot C Ferrand C. IL-1RAP, a key therapeutic target in cancer. Int J Mol Sci. (2022) 23(23):14918. doi: 10.3390/ijms232314918 36499246 PMC9735758

[54] Tian X Yang C Yang L Sun Q Liu N. PTPRF as a novel tumor suppressor through deactivation of ERK1/2 signaling in gastric adenocarcinoma. Oncotargets Ther. (2018) 11:7795–803. doi: 10.2147/OTT.S178152 PMC6223389 30464527

[55] Wojtowicz WM Vielmetter J Fernandes RA Siepe DH Eastman CL Chisholm GB. A human igSF cell-surface interactome reveals a complex network of protein-protein interactions. Cell. (2020) 182:1027–+. doi: 10.1016/j.cell.2020.07.025 PMC7440162 32822567

[56] Heinz S Benner C Spann N Bertolino E Lin YC Laslo P. Simple combinations of lineage-determining transcription factors prime *cis*-regulatory elements required for macrophage and B cell identities. Mol Cell. (2010) 38:576–89. doi: 10.1016/j.molcel.2010.05.004 PMC2898526 20513432

[57] Pang SHM de Graaf CA Hilton DJ Huntington ND Carotta S Wu L. PU.1 is required for the Developmental Progression of Multipotent Progenitors to common lymphoid Progenitors. Front Immunol. (2018) 9. doi: 10.3389/fimmu.2018.01264 PMC6005176 29942304

